# Global Proteomic Analysis Reveals the Roles of MicX in Biofilm Formation and Quorum Sensing in *Vibrio alginolyticus*

**DOI:** 10.3390/foods15061042

**Published:** 2026-03-16

**Authors:** Huan Liu, Qing Liu, Heyang Jiang, Juanjuan Cao, Jiahao Kou, Junjie Liu, Jie Zhao, Jiangwei Wang

**Affiliations:** 1 School of Food Science and Engineering, Shaanxi University of Science & Technology, No. 6 Xuefu Road, Xi’an 710021, China; sheldontsing@outlook.com (Q.L.); jiangheyang1995@163.com (H.J.); 19829264920@163.com (J.C.); k18795016742@163.com (J.K.); 202004020120@sust.edu.cn (J.L.); 18435128287@163.com (J.Z.); w842640005@163.com (J.W.); 2 Shaanxi Research Institute of Agriculture Products Processing Technology, No. 6 Xuefu Road, Xi’an 710021, China

**Keywords:** biofilm, foodborne pathogen, small non-coding RNAs, virulence

## Abstract

*Vibrio alginolyticus* is a foodborne pathogen commonly found in seafood and freshwater products, causing human illness through the consumption of tainted seafood. Small non-coding RNAs (sRNAs) take effect on the stability and translation of their target mRNAs by base-pairing, thereby quickly altering bacterial physiology and pathogenicity at the post-transcriptional level. This work constructed a label-free in-frame deletion mutant and a complement strain of *micX*, a cell-density-associated sRNA in *V. alginolyticus*. The Δ*micX* mutant exhibited reduced growth and a reduction in the synthesis of exopolysaccharides, biofilm, and alkaline serine protease. A TMT-based quantitative proteomic analysis comparing Δ*micX* with the wild-type strain identified 900 differentially expressed proteins, comprising 376 that were upregulated and 524 that were downregulated. The upregulated proteins are primarily associated with porin activity, transmembrane signaling receptor function, and the two-component system. The downregulated proteins are mainly engaged in processes including biofilm formation, cellular communication, and transmembrane transport activity. Of note, the expression levels of proteins involved in the type VI secretion system, exopolysaccharide synthesis, mannose-sensitive hemagglutinin type IV pili (MSHA), and biofilm formation were significantly reduced in the absence of *micX*. Furthermore, the expression levels of proteins associated with quorum sensing (particularly LuxR and AphA) changed significantly in the Δ*micX* vs. WT comparison. These findings strengthened comprehension of the novel sRNA regulatory network and established a theoretical foundation for additional investigations into the virulence of *V. alginolyticus*.

## 1. Introduction

*Vibrio alginolyticus*, a member of *Vibrio* spp., is a foodborne pathogen commonly found in seafood and freshwater products [1]. A study of 502 ready-to-eat (RTE) seafood products collected along the Korean coast reported an overall *Vibrio* spp. prevalence of 23.9%. *V. alginolyticus* was the most frequently detected one across various sample types (17.3%), followed by *V. parahaemolyticus* (11.4%) [2]. Similarly, for the 306 seafood samples collected from Berlin supermarkets (ranging from March 2023 to January 2024), the prevalence of *Vibrio* spp. was 56%. Among the positive samples, *V. alginolyticus* (42%) was the second most prevalent species, just following *V. parahaemolyticus* (58%) [3]. Improperly heated or raw seafood contaminated with *V. alginolyticus* poses a direct health risk, potentially leading to gastroenteritis, sepsis, and other foodborne diseases, threatening human health [1]. The pathogenicity of *V. alginolyticus* is attributed to a variety of virulence factors, such as flagella [4], iron acquisition system [5], type III secretion system (T3SS) [6], type VI secretion system (T6SS) [7], biofilm [8], alkaline serine protease [9], and quorum sensing [10,11]. It is particularly important to note that biofilm is a three-dimensional aggregate formed by bacteria adhering to surfaces and being encapsulated by the extracellular matrix [8], which can protect bacteria from host immunity or antibiotics and enhance the colonization ability of bacteria [8]. And the biofilm formation process involves the participation of MSHA fimbriae (mannose-sensitive hemagglutinin pilus) and VPS (*Vibrio* polysaccharide) in *V. cholerae* [12]. The alkaline serine protease is a major extracellular protease secreted by *V. alginolyticus*. During the process of bacterial infection of the host, it will degrade the host’s tissue proteins, thereby facilitating the invasion and spread of bacteria [9]. Quorum sensing (QS) is an intercellular communication mechanism that enables bacteria to coordinate collective behaviors and represents a key biological process. It involves the synthesis, secretion, and cell-density-dependent detection of extracellular signaling molecules, referred to as autoinducers (AIs) [10]. QS can regulate the expression of various virulence factors, including biofilms and alkaline serine proteases, thereby influencing the pathogenicity of *Vibrios* [10,11].

Small non-coding RNAs (sRNAs), typically ranging from 50 to 300 nucleotides in length, are just transcribed but not translated in prokaryotes. sRNAs have been demonstrated to be closely involved in bacterial virulence, host colonization, stress response, etc., through interaction with their target mRNAs [13]. The sRNA MavR can base-pair with the 3′ region of the *eutR* coding sequence (CDS) and protect it from the degradation from RNaseE, resulting in the maximal ethanolamine-dependent growth. In addition, it also influences motility and intestinal colonization [14]. PinT was the most significantly activated sRNA during *Salmonella enterica* serovar Typhimurium infection. PinT orchestrates the temporal transition from invasion to intracellular replication by directly repressing early virulence effectors and indirectly modulating the SPI-2 virulence island via CRP downregulation [15]. In *V. cholerae*, dozens of regulatory sRNAs were involved in quorum sensing, carbon metabolism, membrane homeostasis, virulence, and biofilm formation [16], etc. The sRNAs Qrr1-4 were indicated to repress the quorum sensing (QS) regulator, HapR, by base-pairing with its ribosome-binding sites [17]. QrrX, a sponge sRNA, regulates QS-controlled phenotypes, such as bioluminescence and biofilm formation, by base-pairing with the 5′ upstream regions of the Qrr1-4 sRNAs [18]. In contrast, the research on sRNA in *V. alginolyticus* is relatively limited. Vvrr1, a *Vibrio* virulence regulatory sRNA, was significantly expressed under stresses, including Cu^2+^, Pb^2+^, Hg^2+^, and low pH in *V. alginolyticus*. In addition, it represses the virulence, biofilm formation, and adhesion by base-pairing with the virulence activator PykF, which encodes pyruvate kinase [19]. sRNA Srvg17985 is a cell-density-dependent sRNA, which inhibited the deamination of L-serine at pH 9.5 and promoted the hydrolysis of X-beta-D-glucuronide, thus affecting the pH stress response [20]. In addition, the absence of *srvg23535* led to stronger resistance to osmotic stress but weaker resistance to pH stress [21]. Qrrs homologous to those in *V. cholerae* were also characterized in *V. alginolyticus* and indicated to regulate QS, virulence, and stress adaptation [22,23,24].

These studies have shown that the regulatory role of sRNAs is widespread in bacteria, and homologous sRNAs may have been extensively investigated in different bacteria. The sRNA MicX was first identified in *V. cholerae*, and it negatively regulates the expression of *vc0972* (encoding an outer membrane protein of unknown function) and *vc0620* (encoding the periplasmic part of the ABC transporter). This sRNA can bind to different target mRNAs at different cell densities [18,25]. MicX was identified to be a core sRNA in *Piscirickettsia salmonis*, and it targets genes including *tolC*, *secA*, *thiC*, etc., involved in processes like membrane transport, pathogenesis, and methionine synthesis [26]. The sRNA MicX was also present in other *Vibrio* species, but no relevant studies have been conducted.

In this study, we aimed to investigate the regulatory role of the conserved sRNA MicX in *V. alginolyticus* virulence. We postulated that MicX is a pleiotropic regulator that affects various virulence-related phenotypes and the molecular pathways that underlie them. To test this hypothesis, we aimed to create a markerless in-frame deletion mutant of the *micX* gene in *V. alginolyticus* EPGS and its complemented strain to figure out the effects of MicX on bacterial phenotypes and global protein profile, providing deeper insights into the regulatory networks controlled by this sRNA.

## 2. Materials and Methods

### 2.1. Bacterial Strains, Plasmids, and Growth Conditions

The strains and plasmids used in this study are listed in Table 1. *V. alginolyticus* strains were normally grown in LBS, which consisted of 3% (*w*/*v*) NaCl in Luria–Bertani (LB) medium, at 30 °C. *Escherichia coli* strains were grown in LB medium at 37 °C. When appropriate, the medium was supplemented with ampicillin (Amp, 100 μg/mL), chloramphenicol (Cm, 25 μg/mL), or L-arabinose (0.04%, *w*/*v*), respectively.

### 2.2. Construction of the ΔmicX Mutant and micX^+^ Strain of V. alginolyticus EPGS

Sequence alignment was conducted using DNAMAN (version 6.0.3.99). The secondary structure of MicX was predicted by the RNAfold Webserver (http://rna.tbi.univie.ac.at//cgi-bin/RNAWebSuite/RNAfold.cgi (accessed on 23 December 2025)). The primers used in this study are shown in Table 2. Using the whole genome of *V. alginolyticus* EPGS as the template, the upstream fragment (628 bp) and downstream fragment (617 bp) of *micX* were amplified by *micX* up-F/R and *micX* down-F/R, respectively. Then, the Δ*micX* fragment was generated by overlap PCR with primers of *micX* up-F and *micX* down-R and introduced into the suicide plasmid, pDM4. Subsequently, the recombinant pDM4-Δ*micX* was successively transformed to *E. coli* DH5α λpir and SM10 λpir, respectively. Through the conjugation between *E. coli* SM10 λpir carrying pDM4-Δ*micX* and the wild-type strain (WT) of *V. alginolyticus*, the Δ*micX* mutant strain was obtained after two rounds of homologous recombination with the positive and negative selection markers on the suicide plasmid, respectively.

For complementation strain construction, the whole open reading frame (ORF) sequence of *micX* was cloned into the plasmid pBAD33. The recombinant plasmid, pBAD33-*micX*, was conjugated into the Δ*micX* mutant strain. Both the mutant strain and the complement strain were characterized by DNA sequencing.

### 2.3. Measurement of Bacterial Growth of WT, ΔmicX and micX^+^

To figure out the functional role of MicX in *V. alginolyticus*, we compared several key phenotypic traits among WT, Δ*micX*, and *micX*^+^. Growth in liquid medium was examined by the measurement of the optical density (OD) of the cell culture at 600 nm. Overnight cultures were collected, diluted to the same cell density (OD_600_ = 1.0) with LBS broth, and then inoculated into the fresh LBS broth. Cultures (3 replicates in each case) were then incubated at 30 °C with continuous shaking at 200 rpm. The OD_600_ was measured at regular time intervals.

### 2.4. Exopolysaccharide Quantification

The quantification of exopolysaccharide (EPS) was determined by the colorimetric phenol–sulfuric acid method described in a previous study, with some modifications [29]. Overnight cultures of WT, Δ*micX*, and *micX*^+^ strains were collected and diluted to the same cell density (OD_600_ = 1.0) and then cultivated in 5 mL fresh LBS broth for another 9 h; then the cell density was measured and recorded as OD_600_. Afterwards, 1 mL of the supernatants of the three types of bacterial cultures was mixed with 3 mL of 100% (*v*/*v*) ethanol and precipitated at 4 °C, respectively. After centrifugation and discarding the supernatant, 1 mL of 6% (*v*/*v*) phenol and 5 mL of concentrated sulfuric acid were added and incubated at room temperature for 20 min. The absorbance of the mixtures at 490 nm was recorded as OD_490_, and the production of EPS was expressed as OD_490_/OD_600_.

### 2.5. Biofilm Assays

Biofilm formation was determined by the crystal violet staining method described in a previous study [30]. In brief, overnight cultures with the same cell density (OD_600_ = 1.0) of WT, Δ*micX*, and *micX*^+^ strains were inoculated (50 μL each) into 15 mL of LBS medium and incubated at 30 °C under static conditions for 24 h, respectively. The unattached cells were washed three times with phosphate-buffered saline (PBS). Then, the attached cells in biofilm were stained with 1% (*w*/*v*) crystal violet, subsequently rinsed, and destained with 33% (*v*/*v*) acetic acid. The optical density of the elution was measured at 570 nm.

### 2.6. Alkaline Serine Protease Activity Assay

The quantitative analysis of alkaline serine protease activity was evaluated following the previously described method with slight modifications [22]. Briefly, the overnight cultures with the same cell density (OD_600_ = 1.0) of WT, Δ*micX*, and *micX*^+^ strains were inoculated into 5 mL fresh LBS medium and cultivated for another 9 h, respectively, and the absorbance of the cell cultures at 600 nm was measured and recorded as OD_6001_. Consequently, 1 mL of the supernatant of each bacterial culture was mixed well with 1 mL of PBS and 0.01 g of Hide Powder Azure (HPA, Sigma-Aldrich, St. Louis, MO, USA). The mixtures were incubated at 37 °C with agitation of 200 rpm for 2 h. After centrifugation at 12,000 rpm for 2 min, the supernatant at 600 nm was detected and recorded as OD_6002_. The alkaline serine protease activity was calculated according to OD_6002_/OD_6001_.

### 2.7. Protein Extraction and TMT Labeling

The WT and Δ*micX* cells, which were collected after being cultured for 9 h, were washed three times with PBS solution, then rapidly frozen in liquid nitrogen and stored at −80 °C for further use. Proteins were extracted using SDT lysis buffer (4% SDS, 100 mM Tris-HCl, 1 mM DTT, pH 7.6) and quantified with a BCA assay. For digestion, the filter-aided sample preparation (FASP) method was applied [31]. Briefly, 200 μg of protein per sample was buffer-exchanged and reduced in UA buffer (8 M urea, 150 mM Tris-HCl, pH 8.0) by repeated ultrafiltration. Then 100 μL of iodoacetamide (100 mM IAA in UA buffer) was added to block reduced cysteine residues, and the samples were incubated for 30 min in darkness, followed by washing with UA and 25 mM NH_4_HCO_3_ buffers. Proteins were then digested overnight at 37 °C with trypsin (4 μg per sample). The resulting peptides were desalted with C18 cartridges, subjected to vacuum drying, and reconstituted in 0.1% formic acid. The peptide concentration was quantified by assessing absorbance at 280 nm. For TMT labeling, 100 μg of peptides from each sample were tagged following the manufacturer’s technique (Thermo Fisher Scientific, Waltham, MA, USA).

### 2.8. High-pH Reversed-Phase Fractionation and LC-MS/MS Analysis

Labeled peptides were fractionated with a High pH Reversed-Phase Peptide Fractionation Kit (Thermo Fisher Scientific, Waltham, MA, USA). The desiccated peptide mixture was reconstituted in 0.1% TFA and subsequently applied to a pre-equilibrated high-pH reversed-phase spin column. Peptides were affixed to the hydrophobic resin in aqueous circumstances, followed by a water wash of the column and desalting using low-speed centrifugation.

Bound peptides were then eluted by applying a step gradient of acetonitrile in a volatile high-pH elution buffer, yielding ten distinct fractions collected by centrifugation. Each fraction was subsequently desalted using C18 cartridges (Empore™ SPE Cartridges C18, standard density; 7 mm bed I.D., 3 mL volume; Sigma-Aldrich, St. Louis, MO, USA) and concentrated under vacuum.

LC-MS/MS analysis of peptide samples was performed using an Easy nLC system coupled online to a Q Exactive mass spectrometer (Thermo Fisher Scientific, Waltham, MA, USA). Separation was performed with a 60 or 90 min gradient. Briefly, the peptides were loaded onto a reverse-phase trap column (Thermo Fisher Scientific, Waltham, MA, USA Acclaim PepMap100, 100 μm × 2 cm, nanoViper C18) connected to the C18-reversed phase analytical column (Thermo Fisher Scientific, Waltham, MA, USA Easy Column, 10 cm long, 75 μm inner diameter, 3 μm resin) in buffer A (0.1% formic acid) and separated with a linear gradient of buffer B (84% acetonitrile and 0.1% formic acid) at a flow rate of 300 nL/min. Mass spectrometry was carried out in positive-ion mode with data-dependent acquisition. Full scan MS spectra (*m*/*z* 300–1800) were acquired at a resolution of 70,000 (at *m*/*z* 200), and the top 10 most intense precursor ions were selected for HCD fragmentation. MS/MS spectra were collected at a resolution of 17,500 (at *m*/*z* 200) with the following settings: automatic gain control (AGC) target 3 × 10^6^, maximum injection time 10 ms, isolation width 2 *m*/*z*, normalized collision energy 30 eV, and dynamic exclusion duration 40 s. The underfill ratio was set to 0.1%, and peptide recognition mode was enabled during the operation.

### 2.9. Protein Analysis

The mass spectrometry raw data for each sample was analyzed using the MASCOT engine (Matrix Science, London, UK; version 2.2) integrated within Proteome Discoverer 1.4 software for identification and quantification purposes. A Student’s *t*-test was conducted on results from three independent experiments, with a *p*-value of less than 0.05 being statistically significant. Proteins exhibiting an absolute expression ratio (Δ*micX*/WT) more than 1.2 were categorized as differentially expressed proteins (DEPs). Functional annotation of all discovered proteins was performed utilizing the Gene Ontology Annotation (GOA) database and the Kyoto Encyclopedia of Genes and Genomes (KEGG) pathway resource. DEPs underwent subsequent enrichment analysis in GO and KEGG. Protein domains were identified using InterProScan via sequence alignment with the Pfam database. Subcellular localization predictions were produced utilizing CELLO, a multi-class support vector machine (SVM)-based framework. Protein sequences were locally analyzed for GO annotation with blastp (version 2.8.0+) and InterProScan (version 5.25-64.0). Homologous sequences were aligned to GO terms and annotated using Blast2GO, with the results depicted by custom R scripts. KEGG Orthology identifiers were allocated by matching protein sequences with the KEGG database, followed by pathway mapping. Enrichment analysis utilized a two-tailed Fisher’s exact test, employing all measured proteins as the background set. *p*-values were adjusted for multiple comparisons via the Benjamini–Hochberg method, with terms exhibiting an adjusted *p*-value < 0.05 deemed significant. Enriched categories were grouped by one-way hierarchical clustering (Euclidean distance, average linkage) utilizing *z*-scores obtained from −log_10_ (*p*-value), with results shown via the gplots R package (version 3.0.3).

### 2.10. Quantitative Real-Time Reverse PCR

For qRT-PCR, cDNA was synthesized from 1 μg of purified RNA using the 5 × All-in-One qRT SuperMix (Vazyme, Xi’an, China). Quantitative real-time PCR (qRT-PCR) was performed with SYBR Premix Ex Taq™ (Tli RNaseH Plus) (TaKaRa, Dalian, China) on a Bio-Rad CFX Opus Real-time PCR system (Bio-Rad Laboratories, Hercules, CA, USA). The primer sequences are provided in Table 2. Each experiment included at least three biological replicates. The relative transcription levels of the target genes were normalized to *gyrB* and calculated using the 2^−ΔΔCt^ method [32].

### 2.11. Data Deposition

The mass spectrometry proteomics data have been deposited to the ProteomeXchange Consortium (https://proteomecentral.proteomexchange.org) via the iProX partner repository [33] with the dataset identifier PXD073542. The username is SheldonTsing, and the password is daydayup123.

### 2.12. Statistical Analyses

All experiments were performed in triplicate. Statistical analyses were performed by GraphPad Prism 9.5.1 (GraphPad Prism, San Diego, CA, USA), and the data are presented as the mean ± SD (*n* = 3). Differences between means were tested by Student’s *t*-test, unless mentioned. Differences were defined as significant at *p* ≤ 0.05.

## 3. Results

### 3.1. Characterization of MicX in Vibrio alginolyticus

**Figure 1:**
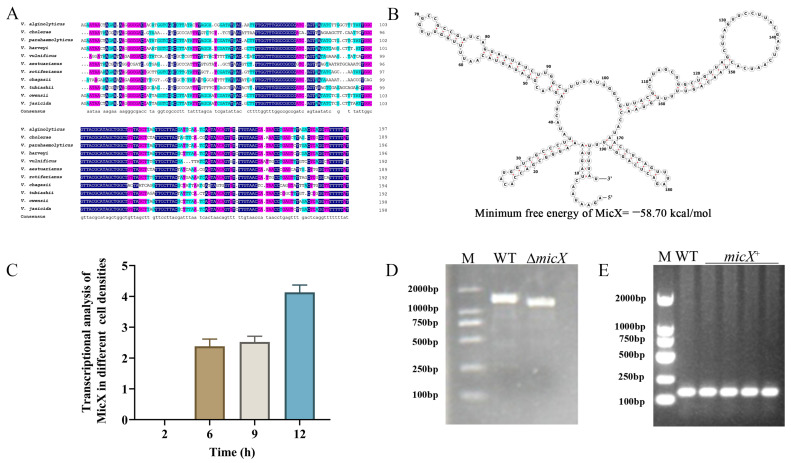
Identification of MicX and construction of mutant and complemented strains in *Vibrio alginolyticus*. (**A**) Sequence alignment of *micX* with homologs from other *Vibrio* species, performed by DNAMAN. (**B**) Secondary structure prediction of MicX by RNAfold. (**C**) Transcript levels of *micX* at different cell densities by qRT-PCR. (**D**) PCR verification of the mutant strain Δ*micX*. (**E**) PCR verification of the complemented strain *micX*^+^.

To investigate the function of MicX in *V. alginolyticus*, an in silico examination was first conducted. Sequence alignment using DNAMAN demonstrated that MicX in *V. alginolyticus* EPGS is phylogenetically conserved, with substantial similarity to the gene in *V. cholerae* (72.22%, Sa5Y_VCA03575) [25], *V. parahaemolyticus* (94.92%, IFVP182_C2MISCRNA13), *V. harveyi* (91.92%, HORM4_MISCRNA46), *V. vulnificus* (74%, VIVU109783_04360), *V. aestuarianus* (68.53%, VAE142_MISCRNA41), *V. rotiferianus* (81.41%, THOE12_MISCRNA19), *V. chagasii* (67.34%, VCHA49P382_MISCRNA1), *V. tubiashii* (74.62%, VITU102760_04025), *V. owensii* (92.96%, THF1D04_MISCRNA8), and *V. jasicida* (91.46%, THF5H11_MISCRNA1); hence, it was termed MicX (Figure 1A). Based on the secondary structure prediction of MicX, the result showed that it can fold into a typical structure featuring several key architectural elements common to functional sRNAs. The structure comprises multiple stem-loops (hairpins), which are critical for stability and function. Several single-stranded regions, particularly in the loops and connecting segments, are also present and these unpaired, accessible sequences are prime candidates for the “seed region”, which would base-pair with the ribosome-binding site or coding region of its target mRNA to exert post-transcriptional regulation (Figure 1B).

Moreover, MicX’s expression profile under different growing phases was detected and the result demonstrated that the expression of MicX is dynamically controlled and strongly impacted by bacterial cell density, implicating a function in quorum sensing or growth-phase-dependent regulatory mechanisms (Figure 1C).

To elucidate the functional role of MicX, an in-frame deletion mutant, *ΔmicX,* was constructed. The successful generation of the mutant was confirmed by the absence of the specific band in the Δ*micX* lane compared to the wild-type (WT) (Figure 1D). To ensure that any observed phenotypes in the mutant were specifically due to the loss of *micX*, the complemented strain *micX*^+^, carrying the recombinant plasmid pBAD33-*micX* in Δ*micX*, was generated and verified by the presence of the specific band of *micX* (Figure 1E).

### 3.2. The Effects of MicX on the Growth of Vibrio alginolyticus

**Figure 2:**
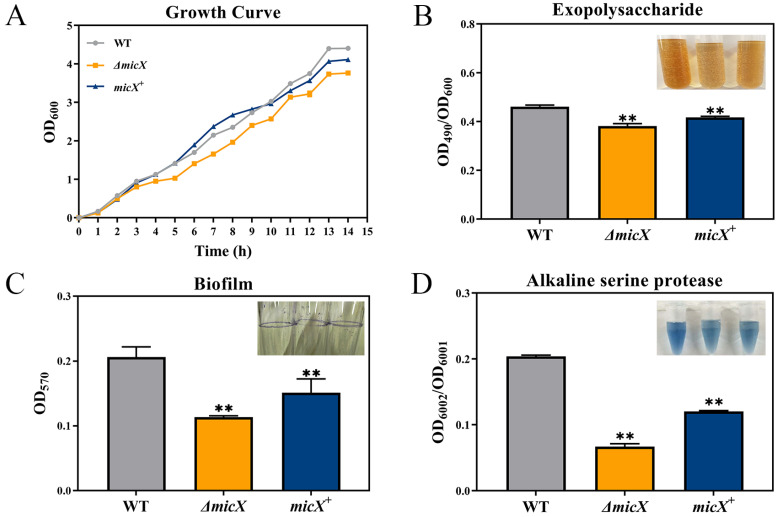
Modulation of MicX on growth, exopolysaccharide production, biofilm formation, and alkaline serine protease activity in *V. alginolyticus*. (**A**) Growth curves of WT, Δ*micX*, and the complemented strain *micX*^+^. (**B**) Quantification of exopolysaccharide production in different strains. (**C**) Biofilm formation in different strains. (**D**) Alkaline serine protease activity in different strains. (** *p* ≤ 0.01).

The deletion of *micX* caused a moderate but noticeable growth defect compared to WT and *micX*^+^, particularly during the exponential stage. Furthermore, the complement of *micX* restored growth to near-WT levels, indicating that MicX is involved in normal growth progression in *V. alginolyticus* (Figure 2A).

### 3.3. Regulation of Exopolysaccharide by MicX in Vibrio alginolyticus

The exopolysaccharide (EPS) assay revealed significant differences among the strains. The production of EPS was markedly impaired in the Δ*micX* mutant, compared to WT (*p* ≤ 0.01) (Figure 2B).

### 3.4. The Role of MicX in Modulating Biofilm Formation of Vibrio alginolyticus

Biofilm formation was also markedly influenced by MicX. The Δ*micX* mutant showed a significantly decreased biofilm-forming capacity relative to WT (*p* ≤ 0.01). This phenotype was reversed in the complement strain, confirming that MicX suppresses biofilm development (Figure 2C).

### 3.5. MicX Promotes the Alkaline Serine Protease Production of Vibrio alginolyticus

Herein, the production of Asp was detected in WT, Δ*micX*, and *micX*^+^ by HPA assay. The Δ*micX* mutant exhibited a significant reduction in Asp production, with an Asp activity of only 33% compared to WT (*p* ≤ 0.01). The complement strain showed an increased Asp production contrast to Δ*micX*, demonstrating that MicX is required for full Asp activity (Figure 2D).

### 3.6. Protein Identification and Quantification and Subcellular Localization Analysis of Differentially Expressed Proteins (DEPs)

**Figure 3:**
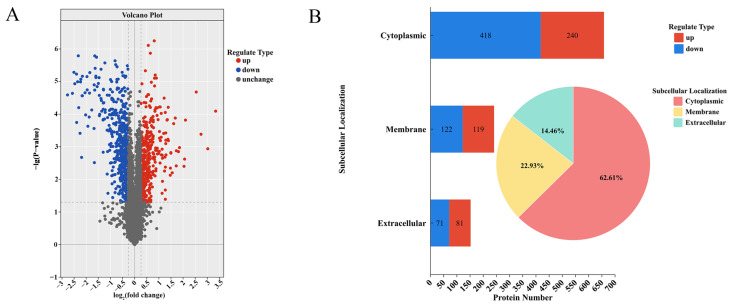
Quantitative and subcellular localization analysis of differentially expressed proteins (DEPs) in the Δ*micX* vs. WT comparison. (**A**) The volcano plot of DEPs. Red and blue points represent significantly upregulated and downregulated DEPs, respectively. The cutoff values used for determining significance are marked on the *y*-axis and *x*-axis. These values are set based on the multiple changes FC > 1.2 or FC < 0.833 and *p* < 0.05. (**B**) Subcellular localization of DEPs. The bar chart (left) and pie chart (right) display the number and proportion of up- and downregulated DEPs, respectively, within each subcellular compartment.

The comparative TMT-based quantitative proteomic analysis showed a total of 617,856 spectra were identified, with 98,683 successfully matched to the database. From these, 37,702 peptides were derived, including 27,971 unique peptides. In sum, 3954 proteins were identified, and 3905 proteins were quantifiable. Proteins with *p* < 0.05 were regarded as significantly expressed. Among them, those with expression multiples > 1.2 were regarded as upregulated proteins, and those with expression multiples < 0.833 were regarded as downregulated proteins. Based on the data, ultimately 900 DEPs were identified in Δ*micX*, compared to WT, with 376 being upregulated and 524 being downregulated (Figure 3A). The findings indicated a widespread proteomic remodeling in response to the loss of *micX*.

CELLO (https://cello.life.nctu.edu.tw/, accessed on 19 February 2025) was used to analyze the subcellular localization of the DEPs [34]. The results revealed that most of the DEPs (658, accounting for 62.61%) were localized in the cytoplasm. In this compartment, 240 proteins were upregulated, and 418 proteins were downregulated. A substantial number of proteins (241, or 22.93%) were connected with the cell membrane, with 119 upregulated and 122 downregulated. In addition, the remaining proteins (152, 14.46%) were projected to be extracellular proteins, with 81 upregulated and 71 downregulated proteins (Figure 3B). This distribution demonstrates the considerable effect of *micX* deletion on the protein composition of several cellular compartments, particularly the cytoplasmic proteome.

### 3.7. Functional Enrichment Analysis of Differentially Expressed Proteins (DEPs)

**Figure 4:**
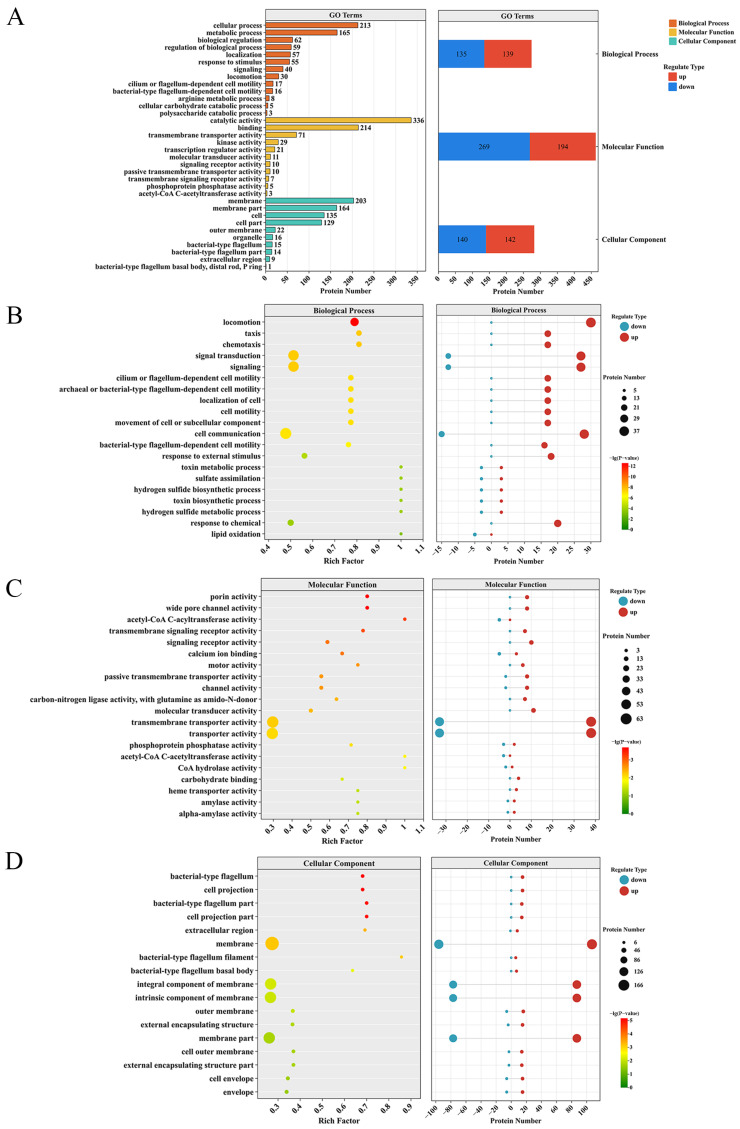
GO enrichment analysis of differentially expressed proteins (DEPs) in the Δ*micX* vs. WT comparison. (**A**) Overall GO annotations of DEPs. The left panel shows the distribution of DEPs across major GO categories, and the right panel details the counts of up- and downregulated proteins within each category. (**B**–**D**) GO term enrichment analysis for the biological process (BP, (**B**)), molecular function (MF, (**C**)), and cellular component (CC, (**D**)) categories. For each category, the left panel presents the enrichment bubble plot, and the right panel shows the corresponding counts of up- and downregulated proteins.

GO functional enrichment analysis was performed on DEPs. All DEPs were annotated in GO, and the number of DEPs was statistically analyzed at the GO secondary functional annotation level. The numbers of DEPs corresponding to BP, MF, and CC were 274, 463, and 282, respectively, among which the upregulated proteins were 139, 194, and 142, respectively, and the downregulated proteins were 135, 269, and 140, respectively (Figure 4A). Under the BP classification, the DEPs show significant changes in processes such as locomotion, chemotaxis, and signal transduction. Upregulated proteins are mainly concentrated in processes such as locomotion (30), localization of cell (17), and chemotaxis (17), while downregulated proteins are mainly concentrated in processes such as lipid oxidation (5), signal transduction (13), and cell communication (15) (Figure 4B). Under the MF classification, porin activity and wide pore channel activity show significant changes. The upregulated proteins mainly focus on functions such as porin activity (8), transmembrane signal receptor activity (7), and signal receptor activity (10), while the downregulated proteins mainly focus on functions such as acetyl-CoA C-acetyltransferase activity (3), acetyl-CoA C-acyltransferase activity (5), and transmembrane transporter activity (33) (Figure 4C). Under the CC classification, bacterial-type flagellum and cell projection show significant changes. The upregulated proteins are mainly focused on membrane (107), cell envelope (15) and cell outer membrane (14), while downregulated proteins are mainly focused on the intrinsic component of membrane (77) and membrane part (77) (Figure 4D).

**Figure 5:**
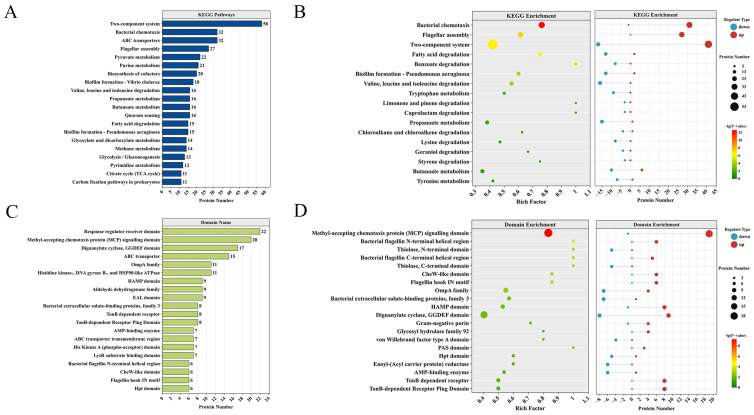
KEGG pathway and protein domain enrichment analysis of differentially expressed proteins (DEPs) in the Δ*micX* vs. WT comparison. (**A**) KEGG pathway annotation of DEPs. (**B**) Enriched KEGG pathways of DEPs. The left panel shows the enrichment bubble plot, and the right panel presents the counts of up- and downregulated proteins in each pathway. (**C**) Protein domain annotation of DEPs. (**D**) Enriched protein domains of DEPs. The left panel displays the enrichment bubble plot, and the right panel indicates the counts of up- and downregulated proteins in each domain category.

We performed a KEGG pathway enrichment analysis on the DEPs to pinpoint their involvement in key biological metabolic processes. The pathways containing the top DEPs in terms of quantity are the two-component system (58), ABC transporters (32), bacterial chemotaxis (32), flagellar assembly (27), and pyruvate metabolism (22) (Figure 5A). KEGG pathway enrichment analysis revealed that significant changes occurred in pathways such as bacterial chemotaxis, flagellar assembly, two-component system, fatty acid degradation, and benzoate degradation (Figure 5B). By classification of various differential proteins, it was found that the upregulated proteins were mainly concentrated in pathways such as bacterial chemotaxis (31), flagellar assembly (27), and two-component system (41). Meanwhile, the downregulated proteins were mainly concentrated in pathways such as valine, leucine and isoleucine degradation (16), propanoate metabolism (15), and biofilm formation (13) (Figure 5B).

Domain enrichment was used to identify protein-level functional units in addition to pathway analysis. The domains with the highest number of DEPs mainly include response regulator receiver domain (22), methyl-accepting chemotaxis protein (MCP) signaling domain (20), diguanylate cyclase, GGDEF domain (17), ABC transporter (15), OmpA family (11), and histidine kinase-, DNA gyrase B-, and HSP90-like ATPase (11). The LysR substrate binding domain and others also contain a certain number of DEPs (Figure 5C). Domain enrichment analysis revealed that the enrichment levels of the methyl-accepting chemotaxis protein (MCP) signaling domain, bacterial flagellin N-terminal helical region, bacterial flagellin C-terminal helical region, and flagellin hook IN motif were relatively high, among which the enrichment level of DEPs under the methyl-accepting chemotaxis protein (MCP) signaling domain was the highest (Figure 5D). All DEPs in the bacterial flagellin N-terminal helical region, bacterial flagellin C-terminal helical region, and flagellin hook IN motif are upregulated proteins (Figure 5D).

### 3.8. MicX Affects Biofilm Formation in Vibrio alginolyticus

**Figure 6:**
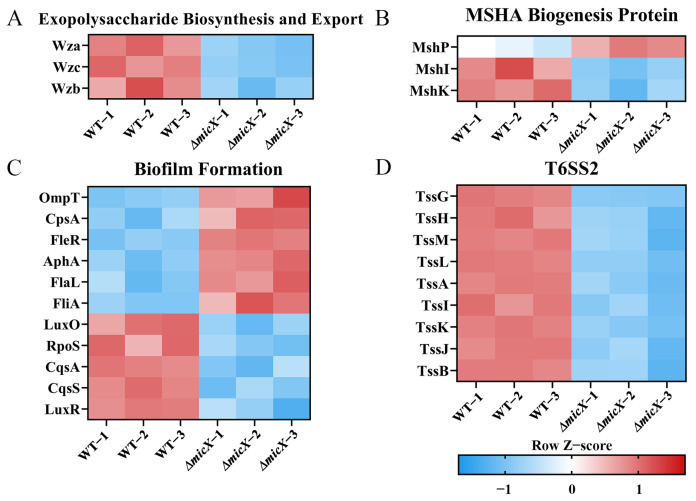
Heat map of the differentially expressed proteins involved in exopolysaccharide production, MSHA biogenesis, biofilm formation, and T6SS2. The expression levels of differentially expressed proteins across different samples are first subjected to a log_2_ transformation, then standardized to Z-scores and presented with different colors in the heat map. Color key indicates standard deviations above (red) or below (blue) the mean expression of each protein. (**A**) DEPs involved in exopolysaccharide production. (**B**) DEPs involved in MSHA biogenesis. (**C**) DEPs involved in biofilm formation. (**D**) DEPs involved in T6SS2.

Among proteins involved in exopolysaccharide synthesis and export, Wza, Wzc, and Wzb decreased 0.56-, 0.55-, and 0.50-fold (FC) compared to wild-type levels, respectively (Figure 6A).

In the MSHA pilus system, MshI and MshK were downregulated 0.82- and 0.68-fold (FC) compared to wild-type levels, respectively, whereas MshP was upregulated by 1.21-fold (FC) (Figure 6B).

Regarding biofilm-related proteins, LuxO, RpoS, CqsA, CqsS, and LuxR were downregulated by 0.78-, 0.67-, 0.61-, 0.59-, and 0.19-fold (FC) compared to wild-type levels, respectively. Conversely, OmpT, CpsA, FleR, AphA, FlaL, and FliA were upregulated by 2.93-, 2.78-, 1.80-, 1.70-, 1.42-, and 1.28-fold (FC), respectively (Figure 6C).

### 3.9. MicX Supports the Type VI Secretion System in Vibrio alginolyticus

All detected proteins associated with T6SS2 showed reduced expression. Specifically, TssG, TssH, TssM, TssL, TssA, TssI, TssK, TssJ, and TssB were downregulated 0.57-, 0.47-, 0.43-, 0.33-, 0.32-, 0.31-, 0.25-, 0.20-, and 0.17-fold (FC) in the Δ*micX* vs. WT comparison, respectively (Figure 6D).

### 3.10. MicX Has an Impact on Quorum Sensing in Vibrio alginolyticus

**Figure 7:**
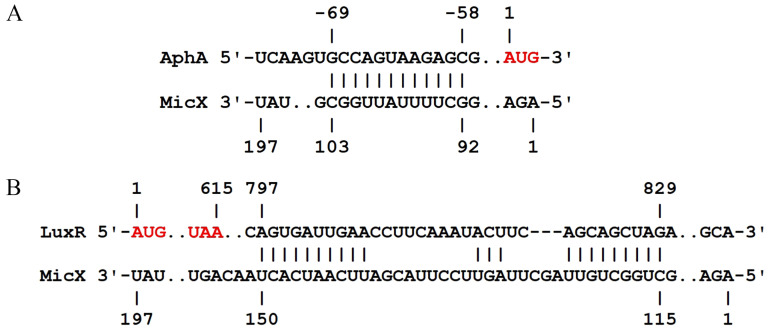
The interaction between MicX and targets predicted by the IntaRNA. (**A**) Predicted interaction region between *aphA* mRNA and MicX. (**B**) Predicted interaction region between *luxR* mRNA and MicX. The translation start codon (AUG) and stop codon (UAA) of the respective mRNAs are indicated in red font. The target mRNA translation initiation site is marked as 1. The upstream and downstream nucleotides are numbered sequentially in negative and positive directions, respectively.

Among the QS-related proteins, the expression levels of LuxO, CqsA, CqsS, and LuxR were downregulated 0.78-, 0.61-, 0.59-, and 0.19-fold (FC) in the Δ*micX* vs. WT comparison, respectively. In contrast, AphA was upregulated by 1.70-fold (FC) (Table 3). The interactions between MicX and the targets were predicted by IntaRNA (http://rna.informatik.uni-freiburg.de/IntaRNA/Input.jsp, accessed on 8 January 2026). Interactions with energy ≤ −8 kcal/mol were regarded as effective. The results indicated that MicX can base-pair with both *luxR* and *aphA* mRNA directly. The specific interaction details between MicX and the targets were depicted in Figure 7. The interaction region between MicX and *aphA* mRNA is located 58–69 nt upstream of the start codon (Figure 7A), and the interaction region between MicX and *luxR* mRNA is located 797–829 nt downstream of the start codon (Figure 7B).

### 3.11. The Proteomics Results Were Verified by qRT-PCR

**Figure 8:**
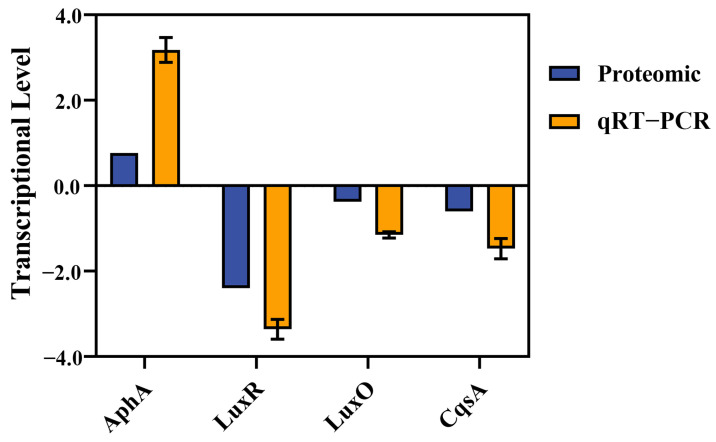
Validation of proteomics results by qRT-PCR. The mRNA expression levels of four genes encoding differentially expressed proteins were analyzed by qRT-PCR and compared with their corresponding protein abundance changes from proteomics. Gene expression was normalized to *gyrB*. Blue and orange columns represent the relative abundance from proteomics and qRT-PCR, respectively.

Four genes were selected for the qRT-PCR assay. The results demonstrated that the transcriptional levels of *luxR*, *luxO*, and *cqsA* were downregulated by 3.36-fold, 1.15-fold, and 1.47-fold, respectively, in the mutant strain compared to the wild-type strain. Whereas the transcriptional level of *aphA* was upregulated by 3.18-fold in Δ*micX* vs. WT comparison (Figure 8). These findings are consistent with the proteomic data (Table 3).

## 4. Discussion

Small non-coding RNAs (sRNAs) play a crucial regulatory role in bacteria, especially in pathogenic bacteria. Through sophisticated post-transcriptional regulatory mechanisms, they control the expression of virulence factors, assist pathogenic bacteria in rapidly adapting to environmental alteration, and so on. MicX, a small RNA initially characterized in *V. cholerae*, acts as a repressor of the outer membrane protein (VC0972) and the periplasmic component of a peptide ABC transporter (VC0620). Notably, its mRNA targeting specificity varies with changes in cell density [18,25]. In *P. salmonis*, MicX is recognized as a core sRNA, regulating multiple genes, such as *tolC*, *secA*, and *thiC*, that participate in biological functions including transmembrane transport, pathogenicity, and methionine biosynthesis [26]. MicX was highly conserved in *Vibrio* species, and the gene in *V. alginolyticus* EPGS shared substantial similarities to that in *V. cholerae* (72.22%) [25], *V. parahaemolyticus* (94.92%), *V. harveyi* (91.92%), and *V. vulnificus* (74%), respectively (Figure 1A), indicating that MicX is an important sRNA performing critical functions and potentially participating in the core regulatory network in *Vibrio*. In this study, we constructed a markerless in-frame deletion mutant strain, Δ*micX*, and a complemented strain, *micX*^+^, and elucidated the global roles of MicX in *V. alginolyticus* by TMT-based quantitative proteomics assay.

### 4.1. MicX Positively Regulates Biofilm Formation Through Multiple Pathways

The absence of *micX* caused a significant reduction in the biofilm formation in *V. alginolyticus*, with a 50% decrease in the Δ*micX* vs. WT comparison. Biofilm refers to a structured community where microbial cells are firmly attached to a surface and embedded in a biofilm matrix composed of extracellular polymers [35]. Biofilm formation is a dynamic process, which mainly consists of three parts: surface adhesion of bacteria, assembly of biofilm substrates, and diffusion of biofilms [36]. In *V. cholerae*, free cells approach the surface through flagella, and the bacteria adhere to the surface mediated by MSHA and adhesion proteins (such as FrhA and CraA). Then, the bacteria use VPS (*Vibrio* polysaccharide) as the skeleton and mediate the cross-linking between cells with the matrix proteins secreted by the type II secretory system (such as RbmA and RbmC) [36]. Meanwhile, extracellular DNA (eDNA) and outer membrane vesicles (OMVs) will also be embedded in it. These substances jointly construct the three-dimensional structure of the biofilm. When the biofilm matures, proteases will degrade the above-mentioned proteins.

Exopolysaccharide (EPS) is a high-molecular-weight polymer secreted outside cells. As the structural matrix of biofilms, it is crucial for bacterial attachment, aggregation, and resistance to environmental stress [36]. In this study, the expression levels of Wza, Wzc, and Wzb in Δ*micX* decreased 0.56-, 0.55-, and 0.50-fold (FC) compared to WT, respectively. The synthesis and export of EPS is under the joint regulation of Wza, Wzb, and Wzc [37,38]. Wza is a transmembrane transporter that can directly interact with the periplasmic domain (Motif 3) of Wzc to form a hydrophilic channel for the export of polysaccharides [37]. Wzc, which is a tyrosine kinase, can be phosphorylated, and the phosphorylated Wzc affects the formation of the hydrophilic channel and thus influences the export of EPS. Specifically, the export of EPS was inhibited by phosphorylated Wzc and promoted by dephosphorylated Wzc. As a tyrosine phosphatase, Wzb mediates the dephosphorylation of Wzc to promote the export of EPS [37,38]. In *V. anguillarum*, the EPS production and bacterial virulence of the *wza*-deficient strain are significantly lower than those of the wild-type strain and the biofilm formation ability of the mutant is nearly lost [39]. Therefore, the downregulation of Wza, Wzc, and Wzb expression levels may lead to the reduction in polysaccharide production and transport, thereby affecting the biofilm formation of Δ*micX*.

MSHA fimbriae (mannose-sensitive hemagglutinin pilus) are type IV fimbriae existing on the surface of various *Vibrio* bacteria. Their core function is to act as an important adhesin, mediating the initial adhesion of bacteria to various biological and abiotic surfaces [12]. This attachment is the crucial first step for *Vibrio* to establish infection and form biofilm [40]. According to the results of proteomics, MshI and MshK decreased 0.82- and 0.68-fold (FC) in the Δ*micX* vs. WT comparison, whereas MshP was upregulated by 1.21-fold (FC). In *V. cholerae*, the biosynthesis of MSHA fimbriae involves the joint participation of multiple genes, which are distributed in the secretory operon and the structural operon. Among them, MshI, MshK, and MshP may be involved in the secretion or assembly of MSHA fimbriae proteins [41]. Additionally, in *Aeromonas veronii*, the deletion of the *mshK* gene significantly reduces the ability of biofilm formation and inhibits the expression of other MSHA genes [42]. It can be inferred that MicX may promote biofilm formation by increasing the synthesis of MSHA fimbriae through the upregulation of MshK and MshI in *V. alginolyticus*.

### 4.2. MicX Is Required for T6SS2 Assembly and Function

Bacteria inject effector proteins into neighboring bacteria or eukaryotic cells through the T6SS, thereby gaining an advantage in environmental competition and host colonization processes [43,44]. The proteomic results of this study indicated that, compared with WT, the expression of TssA and TssB, which are involved in the assembly of the tail tube/sheath complex; TssG, TssI, and TssK, which are involved in the composition of the baseplate complex; TssJ, TssL, and TssM, which are involved in the composition of the membrane complex; and TssH, which is responsible for sheath recycling, were all significantly downregulated in Δ*micX* (Figure 6D). T6SS is mainly composed of three parts: the membrane complex, the baseplate complex, and the tail tube/sheath complex. The baseplate complex, consisting of TssE, TssF, TssG, TssK, and VgrG (TssI), is responsible for loading the effector proteins. The membrane complex, composed of TssJ, TssL, and TssM, connects with the baseplate complex and plays a role in stabilizing T6SS. Additionally, the tail tube/sheath complex is composed of proteins such as TssA, TssB, TssC, and Hcp. The contraction of the sheath tube will drive the effector proteins into the target cell, and then ClpV (TssH) recovers the sheath and reassembles it [45]. There are two T6SS gene clusters in *V. alginolyticus*, namely T6SS1 and T6SS2, among which T6SS2 is the main bacterial killing system [43,46]. In *V. parahaemolyticus*, T6SS2 affects biofilm formation and cell adhesion [45], and the deletion of *icmF2* (*tssM2*) showed a significant decrease in biofilm formation [47]. These findings suggest that the absence of MicX inhibited the assembly of T6SS2, thereby affecting the biofilm formation and bacterial competence of *V. alginolyticus*.

### 4.3. MicX Modulates the Quorum Sensing Circuit by Regulating LuxR and AphA

Our proteomic analysis revealed that the absence of MicX significantly altered the expression of key quorum sensing elements. Compared to WT, Δ*micX* exhibited downregulation of LuxO (0.78-fold), CqsA (0.61-fold), CqsS (0.59-fold), and LuxR (0.19-fold), while AphA was upregulated by 1.70-fold (Table 3). Furthermore, the sRNA-mRNA interaction predictions using IntaRNA indicated the direct base-pairing interactions between MicX and both LuxR and AphA mRNAs (Figure 7).

Quorum sensing (QS) is a chemical communication process used by bacteria to coordinate group behavior and is an important biological behavior. QS involves the generation, release, and population-wide detection of extracellular signaling molecules called autoinducers (AI). The behaviors controlled by QS include bioluminescence, virulence factor production, biofilm formation, etc. This process is ineffective when carried out by individual bacteria, but it will play a role when carried out synchronously by a population [10]. In *Vibrios*, AI is generally divided into three major categories, namely AI-1 produced by LuxM, CAI-1 produced by CqsA, and AI-2 produced by LuxS, and each of them is detected by the receptors LuxN, CqsS, and LuxPQ, respectively [10,48]. When AIs are deficient, these receptors act as kinases to transfer phosphate groups to LuxU and subsequently to LuxO [10]. The phosphorylated LuxO, together with sigma factor σ^N^, will jointly induce the expression of four sRNAs, which are named Qrr1-4 [17]. sRNA Qrr1-4 regulates gene expression through base-pairing with target mRNA [18] with the assistance of the Hfq protein [17]. At this time, they will promote the expression of the low-cell-density master regulator, AphA [18], and inhibit the expression of the high-cell-density master regulator, LuxR [10]. In high cell density, AIs will bind to the corresponding receptors, and the receptors no longer function as kinases. In this case, AphA will no longer be activated, and LuxR will be activated [10]. LuxR/HapR-type transcriptional regulatory proteins are the core transcriptional regulatory factors of all pathogenic *Vibrio* under high cell density and can affect their motility, extracellular protease synthesis, biofilm formation, secretory system, etc. [11]. In *V. harveyi* and *V. cholerae*, LuxR/HapR can activate the expression of genes related to motility and extracellular proteases, demonstrating a high level of motility and extracellular protease synthesis ability at high cell density [11]. LuxR positively regulates the biofilm matrix development of *V. alginolyticus* [49] and directly activates the expression of the alkaline serine protease, Asp [9]. As a key regulator in low cell density, AphA can bind to the promoter region of LuxR to inhibit the expression of LuxR [50], thereby negatively regulating the production of Asp [51]. The expression of LuxR was downregulated by 3.36-fold, while AphA was upregulated by 3.18-fold in the Δ*micX* vs. WT comparison. The upregulation of AphA inhibited the expression of LuxR, which in turn led to a decrease in the production of Asp and biofilm formation in Δ*micX.* Of note, the base-pairing interaction between the target mRNA and MicX (Figure 7) indicated that MicX may change the target’s conformation by binding to the 5′ UTR of *aphA* mRNA, thereby inhibiting the binding of ribosomes and affecting translation [52]. Additionally, MicX may protect *luxR* mRNA from being degraded by RNase by occupying the 3′ UTR of *luxR* mRNA, finally promoting the expression of LuxR [53]. The underlying regulatory mechanism of MicX on LuxR and AphA will be discovered in future work. In summary, MicX may be involved in the quorum sensing processes, biofilm formation, and T6SS2 in *V. alginolyticus* through action on the expression of LuxR and AphA.

## 5. Conclusions

The cell-density-associated sRNA MicX plays pleiotropic roles in *V. alginolyticus*, such as bacterial growth, biofilm formation, alkaline serine protease production, and so on. A global proteomic assay further substantiated these findings, identifying 900 DEPs upon *micX* deletion. The significant downregulation of proteins involved in key virulence-associated pathways, including T6SS2, exopolysaccharide biosynthesis, MSHA pilus synthesis, and biofilm formation. Moreover, MicX was involved in the quorum sensing process and cell-density-dependent behaviors through the two key quorum sensing regulators, such as LuxR and AphA.

## Figures and Tables

**Table 1:** Strains and plasmids used in this study.

Strain or Plasmid	Relevant Characteristics	Reference or Source
*Escherichia coli*		
DH5α λpir	Host for π-requiring plasmids	Lab collection
SM10 λpir	Host for π-requiring plasmids, conjugal donor	Lab collection
*Vibrio alginolyticus*		
EPGS	Wild-type, isolated from the aquiculture farm of the South China Sea, Amp^r^	CCTCC No. AB 209306
Δ*micX*	EPGS, in-frame deletion in *micX*, Amp^r^	This study
*micX* ^+^	EPGS, Δ*micX* complemented with intact *micX* gene, Amp^r^, Cm^r^	This study
Plasmids		
pDM4	Suicide vector, pir-dependent, R6K, SacBR, Cm^r^	[27]
pBAD33	Arabinose-induced expressing vector, Cm^r^	[28]

Note: Amp^r^ represents ampicillin-resistant, Cm^r^ represents chloramphenicol-resistant.

**Table 2:** Nucleotide sequences of primers used in this study.

Primer	Sequence (5′-3′)
*micX* up-F	GTCGACTTACGCGTTTGCTCGTAT
*micX* up-R	AACCTGAGTCCCATGTGGTCGCCCTTTTCT
*micX* down-F	GACCACATGGGACTCAGGTTTTTTTATGCC
*micX* down-R	GAGCTCGAAGAAATGGAAGCACACGT
*micX* com-F	TCGCCCTTATACTTAGCACGGA
*micX* com-R	TAAACTCAGGTTATGGTTA
M13-F	TGTAAAACGGCCAGT
M13-R	CAGGAAACAGCTATGACC
q*micX* -F	TCGCCCTTATACTTAGCACGG
q*micX* -R	CAGCTATGCGTAACGCCAAT
q*aphA*-F	CTTAACTGTTCTTAGCACTCGC
q*aphA*-R	TGTTGAGTTCACGGTACACCT
q*luxR*-F	CACGCGAAGACTTGGTGGAT
q*luxR*-R	TGACAGTCTTGGCTGACAAGCT
q*luxO*-F	GGCTCTGTACCGCTCATACC
q*luxO*-R	AATGGCATCCCTACCTGTGC
q*cqsA*-F	GCCGGAGCACAAATTCATCC
q*cqsA*-R	CACGTAGAGGAGCGATGGTC
*gyrB*-F	TAGCGACGGCTCATACGTTC
*gyrB*-R	CACTGGCTGGTGAAGCACTA

Note: The underlined ones are homologous arm sequences.

**Table 3:** The differentially expressed proteins involved in quorum sensing.

Gene ID	Protein Name	Protein Description	FC (Δ*micX*/WT)	*p*-Value
EH99_RS20865	AphA	PadR family transcriptional regulator	1.70	0.000171883
EH99_RS05580	LuxO	quorum sensing sigma-54-dependent transcriptional regulator LuxO	0.78	0.000727886
EH99_RS00420	CqsA	alpha-hydroxyketone-type quorum sensing autoinducer synthase	0.61	0.000401385
EH99_RS00425	CqsS	response regulator	0.59	0.000179867
EH99_RS09275	LuxR	HTH-type transcriptional regulator LuxR	0.19	0.000179296

## Data Availability

The original contributions presented in this study are included in the article/Supplementary Material. Further inquiries can be directed to the corresponding author.

## Supplementary Materials

The following supporting information can be downloaded at https://www.mdpi.com/article/10.3390/foods15061042/s1, Table S1: The information about the differentially expressed proteins in Δ*micX*/WT.
