# Supplementary material for: Global Proteomic Analysis Reveals the Roles of MicX in Biofilm Formation and Quorum Sensing in Vibrio alginolyticus
Source: Foods. 2026 Mar 16;15(6):1042. doi: 10.3390/foods15061042 (PMC13025180; doi:10.3390/foods15061042)
Supplement: Supplementary file 1 [file foods-15-01042-s001.zip › foods-4162370-supplementary.pdf]

Table S1. The information about the differentially expressed proteins in Δ*micX*/WT

Gene ID	Protein Name	Protein Description	FC (Δ*micX* / WT)	*p*-value
**Exopolysaccharide biosynthesis and export**				
EH99_RS10935	Wza	polysaccharide export protein	0.56	0.005997035
EH99_RS10925	Wzc	polysaccharide biosynthesis tyrosine autokinase	0.55	0.000351834
EH99_RS10930	Wzb	low molecular weight protein-tyrosine-phosphatase	0.50	0.003152525
**MSHA biogenesis protein**				
EH99_RS18635	MshP	MSHA biogenesis protein MshP	1.21	0.024972431
EH99_RS18565	MshI	MSHA biogenesis protein MshI	0.82	0.001360114
EH99_RS18575	MshK	MSHA biogenesis protein MshK	0.68	0.000330318
**Biofilm Formation**				
EH99_RS03405	OmpT	outer membrane protein OmpT	2.93	0.006267943
EH99_RS11920	CpsA	capsular polysaccharide synthesis enzyme CpsA	2.78	0.004510611
EH99_RS04715	FleR	sigma-54-dependent Fis family transcriptional regulator	1.80	6.3035E-06
EH99_RS20865	AphA	PadR family transcriptional regulator	1.70	0.000171883
EH99_RS04710	FlaL	flagellar sensor histidine kinase FlaL/FleS	1.42	0.000975823
EH99_RS04815	FliA	RNA polymerase sigma factor FliA	1.28	0.001771226
EH99_RS05580	LuxO	quorum-sensing sigma-54 dependent transcriptional regulator LuxO	0.78	0.000727886
EH99_RS20620	RpoS	RNA polymerase sigma factor RpoS	0.67	0.001596029
EH99_RS00420	CqsA	alpha-hydroxyketone-type quorum-sensing autoinducer synthase	0.61	0.000401385
EH99_RS00425	CqsS	response regulator	0.59	0.000179867
EH99_RS09275	LuxR	HTH-type transcriptional regulator LuxR	0.19	0.000179296
**Type VI secretion system**				
EH99_RS10255	TssG	type VI secretion system baseplate subunit TssG	0.57	2.30395E-06
EH99_RS10250	TssH	type VI secretion system ATPase TssH	0.47	0.000324297
EH99_RS10305	TssM	type VI secretion system membrane subunit TssM	0.43	7.35618E-05
EH99_RS10310	TssL	type VI secretion system protein TssL, long form	0.33	6.82547E-06
EH99_RS10290	TssA	type VI secretion system protein TssA	0.32	1.82233E-05
EH99_RS10240	TssI	type VI secretion system tip protein TssI/VgrG	0.31	0.000274586

EH99_RS10315	TssK	type VI secretion system baseplate subunit TssK	0.25	7.03825E-06
EH99_RS10320	TssJ	type VI secretion system lipoprotein TssJ	0.20	6.30085E-05
EH99_RS10285	TssB	type VI secretion system contractile sheath small subunit TssB	0.17	2.29686E-05
